# Doxorubicin as a Potential Treatment Option in Canine Mammary Tumors

**DOI:** 10.3390/vetsci10110654

**Published:** 2023-11-14

**Authors:** Madalina Luciana Gherman, Oana Zanoaga, Liviuta Budisan, Lajos Raduly, Ioana Berindan-Neagoe

**Affiliations:** 1 Experimental Center, Iuliu Hatieganu University of Medicine and Pharmacy, 400349 Cluj-Napoca, Romania; luciana.gherman@umfcluj.ro; 2 Research Center for Functional Genomics, Biomedicine and Translational Medicine, Iuliu Hatieganu University of Medicine and Pharmacy, 400337 Cluj-Napoca, Romania; oana.zanoaga@umfcluj.ro (O.Z.); liviuta.petrisor@umfcluj.ro (L.B.); ioana.neagoe@umfcluj.ro (I.B.-N.)

**Keywords:** cancer, canine, mammary tumors, epithelial to mesenchymal transition, doxorubicin

## Abstract

**Simple Summary:**

Due to the limited options in mammary tumor treatments, the following study demonstrates the efficiency of Doxorubicin on canine mammary tumor therapy and in the alteration of expression of the key genes involved in the epithelial-to-mesenchymal transition processes.

**Abstract:**

Canine mammary tumors represent one of the leading malignant pathologies in female dogs, displaying the importance of efficient therapeutic findings, besides the golden-standard surgery, able to limit the development of the disease. Studies in human cancers demonstrated that Doxorubicin presents a good effect in different biological processes like apoptosis, autophagy, the cell cycle, cell invasion, and the epithelial-to-mesenchymal transition. This study followed the effects of Doxorubicin on two canine mammary cancer cell lines P114 and CMT-U27. Doxorubicin treatment in both cell lines shows an inhibitory effect in cell proliferation and an alteration in expression of the EMT-related genes. The obtained results provide valuable information for revealing the link between Doxorubicin, phenotypic changes, and proliferation dynamics in canine mammary tumor models.

## 1. Introduction

Mammary tumors represent the most frequent neoplasia in female dogs. In epidemiological studies, the annual incidence of mammary tumors is 40–50% for benign and almost 50% for malignant tumors [1,2,3,4]. In the countries like the USA, mammary tumors in canines have decreased because of early neutering, but the most frequent mammary cancer forms in female dogs are still present in most of the European countries [1,5]. The golden-standard treatment procedure worldwide is represented by the surgical excision of the mammary chain but with unsatisfactory results. A high number of dogs with mammary cancer show lymphatic or vascular invasion and high rates of recurrence and metastasis [6,7,8,9]. There is limited information about chemotherapy and radiotherapy efficacy in treating canine mammary tumors (CMTs), probably because the procedures are expensive. Despite some case reports regarding the responses to chemotherapeutics such as Doxorubicin [10], mitoxantrone [11], paclitaxel [12,13], and carboplatin [4], it is not clear if chemotherapy improves the overall survival of the treated dogs [5,8,14].

One of the most used therapeutic agents in human cancer is the anthracycline Doxorubicin (DOXO) [15,16,17]. In recent years, a few studies have shown the efficiency of this drug in canine patients [7,18]. Doxorubicin has been reported by many studies to treat cancers including breast, lung, thyroid, and hematologic cancers [19,20]. Doxorubicin induces the production of free radicals, intercalation into DNA strands and inhibition of topoisomerases I and II, causing DNA damage and activating caspases, ultimately leading to apoptosis [16,21]. 

The epithelial-to-mesenchymal transition (EMT) is essential in embryonic development and tissue regeneration. The EMT is characterized by a progressive loss of the cells’ epithelial characteristics, which acquire mesenchymal features through a molecular reprogramming of the cells, promoting progression and invasion of the neighboring stroma [22,23,24]. The EMT has been demonstrated to play a key role in the development of the metastatic process in canine mammary carcinoma, promoting the idea that the EMT is a dynamic reversible process [25]. An aberrant reactivation of the EMT is associated with malignant properties of tumor cells during cancer progression and metastasis, including migration and invasiveness, increased tumor stemness, and enhanced resistance to therapy [26,27,28]. Studies performed in humans identified the EMT as involved in DOX-resistance to therapy, stimulating the migration of tumor cells and metastasis; furthermore, the EMT mediates the resistance of cancer cells to DOX-mediated apoptosis [28,29].

The present study aimed to analyze the inhibitory effect of Doxorubicin in vitro models of canine mammary cancer to evaluate the efficiency of this therapeutic agent and the expression levels of key genes involved in the EMT process.

## 2. Materials and Methods

### 2.1. Cell Lines and Cell Culture

This study was conducted in two canine mammary carcinoma cell lines (P114 and CMT-U27) kindly donated by Prof. Gerard Rutteman from the Netherlands and Prof. Eva Hellmen from Sweden. The two canine cell lines were cultured in RPMI supplemented with 10% Fetal Bovine Serum (FBS) and 1% Glutamine for P114, and in low-glucose DMEM supplemented with 10% Fetal Bovine Serum (FBS) and 1% Glutamine for CMT-U27. The culture medium and supplements were obtained from Sigma-Aldrich (St. Louis, MO, USA). Both cell lines were treated with Doxorubicin (2.5 µM) for 48 h and different in vitro functional tests were performed.

The P114 cell line is a canine anaplastic mammary carcinoma (AMC) cell line shown in dogs and felines. In dogs, AMC is considered the most aggressive type of mammary tumor, showing a low frequency, and is associated with a short survival time. The CMT-U27 cell line is a canine mammary carcinoma cell line [30,31,32].

### 2.2. MTT Cell Viability Assay

A total of 1 × 10^4^ cells/well were cultured in 96-well culture plates for 24 h at 37 °C in 5% CO_2_ atmosphere incubators. After 24 h incubation, cell cultures were treated with Doxorubicin (DOXO) doses (Sigma Aldrich, St. Louis, MO, USA). At 48 h after treatment, the medium was discarded, and 100 µL MTT solution was added to every well. After 2 h incubation at 37 °C, the MTT solution was removed, and the formazan crystal was solubilized with 100 µL DMSO (dimethyl sulfoxide) (Sigma-Aldrich). The absorbance was measured at 570/690 nm for the cell viability assay in a microplate reader (Synergy H1 Hybrid Reader Biotech).

### 2.3. Apoptosis Assay through Fluorescence Microscopy

The Multi-Parameter Apoptosis Kit (Cayman, Ann Arbor, MI, USA) was used for the fluorescence microscopy evaluation of apoptosis. Cell staining was performed according to the manufacturer’s protocol with tertamethylrhodamin ethyl ester (TMRE) for mitochondria and Hoechst for nuclei. The stained cells were analyzed at UV wavelengths for Hoechst and 560/595 nm for TMRE staining on an Olympus IX71 inverted microscope. Hoechst is a specific stain for nucleus morphology and counting, while TMRE indicates the mitochondrial membrane activity potential.

Apoptosis evaluation was also performed through triple-fluorescence staining using Actin-stain 488 Fluorescent Phalloidin (Cytoskeleton, Inc., Denver, CO, USA) for cytoskeleton, Mitotracker (Invitrogen) for mitochondria labeling, and DAPI (4′,6-diamidino-2-phenylindole) (Invitrogen) for nucleus staining.

### 2.4. Confluency Assay

Cell lines were cultured in a 96-well culture plate for 48 h and then treated with 2.5 µM DOXO. After 48 h treatment, cell confluency was analyzed using a Celigo Image Cytometer (Nexcelom, Lawrence, MA, USA).

### 2.5. Cell Cycle Assay using Celigo

Cell lines were cultured in a 96-well culture plate for 48 h and then treated with 2.5 μM DOXO. After 48 h treatment, cells were fixed in ice-cold methanol at 4 °C for 30 min. After fixation, the cells were marked with RNase A and Propidium Iodide (PI) solution for 45 min in the dark at 37 °C in 5% CO_2_ atmosphere incubators. The cell cycle analysis was performed using Celigo Image Cytometer (Nexcelom).

### 2.6. Colony Formation Assay

P114 and CMT-U27 cells were incubated with 2.5 µM Doxorubicin for 48 h to induce DNA damage. The cells were detached after 48 h, counted, and reseeded in the standard medium in triplicate for each condition and cellular type (500 cells/6 well plate). Cells returned to the incubator for 7 days to form colonies and then were fixed with ice-cold methanol for 15 min, dried, and stained with 0.5% crystal violet in 25% methanol for 20 min. After washing with water and drying, colonies were counted and visualized using the Celigo Image Cytometer (Nexcelom).

### 2.7. Scratch Assay

Canine mammary cancer cells were individually pre-treated with DOXO for 48 h in 24-well plates. After treatment, cell proliferation was blocked through a 30 min pretreatment with mitomycin C (40 μg/mL). A scratch was then made in each well using a 20 μL pipette tip, and cells were maintained in a medium with 1% serum. The gaps were scaled (µm^2^) at 0, 8, and 24 h and visualized using an inverted microscope Olympus IX71.

### 2.8. Cell Invasion Assay

Canine cells (15 × 10^3^ cells/well) were cultured in the upper 8 µm Falcon cell culture inserts pre-coated with 100 µL Matrigel in 500 µL media without serum. In the lower chambers (24 well plates), 750 µL complete medium containing 10% FBS was added. After 48 h of incubation, the cells that had invaded through the pores and attached to the bottom surface of the membrane were stained with Calcein-AM (Invitrogen, Waltham, WA, USA) according to the manufacturer’s protocol. Images of the stained invaded cells were captured under an Olympus IX71 inverted fluorescent microscope.

### 2.9. Gene Expression Evaluation Using PCR Array

For the extraction of total RNA, the Trizol (TriReagent Sigma-Aldrich) protocol was used, and the quantitative and qualitative control was performed using the Nanodrop-1000 spectrophotometer (Thermo Scientific, Waltham, WA, USA) and the Agilent Bioanalyzer 2100. cDNA synthesis was performed using the RT^2^ First Strand Kit (Qiagen, Singapore) from 1000 ng of total RNA, according to the manufacturer’s recommendations. For the RT^2^ Profiler PCR Array (PAFD-090ZE-1 Dog Epithelial-to-Mesenchymal Transition—EMT-Qiagen), we used the SYBR Green Master Mix and the ViiA7 instrument from Applied Biosystems according to the manufacturer’s protocol. Alterations in gene expression levels for the studied genes were evaluated using the Qiagen RT^2^ Profiler PCR Array and Assays Data Analysis software (v3.5).

### 2.10. Statistical Analysis

Standard deviation (mean ± SD) and a *t*-test were used to study the difference between experimental and control conditions (*p*-value < 0.05 was considered statistically significant; ns/*p* > 0.05; */*p* ≤ 0.05; **/*p* ≤ 0.01; ***/*p* ≤ 0.001; ****/*p* ≤ 0.0001). Statistical analyses and graphic representation were performed using GraphPad Prism software (version 8.0; GraphPad Software, Inc., San Diego, CA, USA).

## 3. Results

### 3.1. Doxorubicin Inhibits Cell Viability in Both In Vitro Canine Mammary Cancer Models

**Figure 1:**
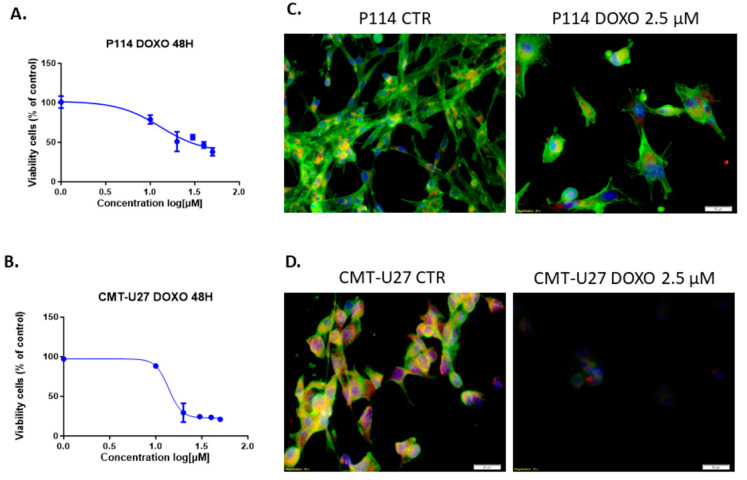

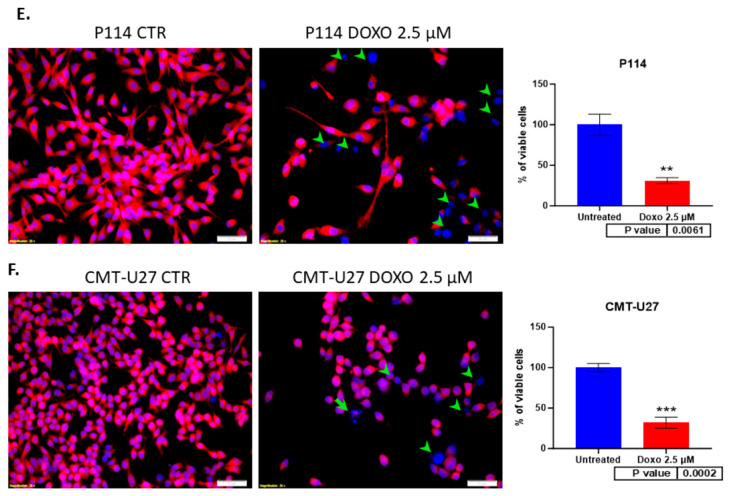
MTT cell viability assay and IC50 value identification after treatment with gradual doses of Doxorubicin in P114 and CMT-U27 canine mammary cancer cell lines for 48 h. Doxorubicin inhibits cell viability within all cell lines with an IC50 of 13 µM for P114 and 13.80 µM for CMT-U27 cell line (**A**,**B**). Actin damage and mitochondrial membrane potential activity and nuclear morphology evaluation. Observation of the Phalloidin-FITC/Mitotracker/DAPI staining using Olympus IX71 inverted fluorescence microscope after 48 h of treatment with 2.5 µM of Doxorubicin (image magnification 20×) (**C**,**D**), and the mitochondrial potential activity inhibition, cytoskeleton damage, and nuclear morphology changes using TMRE/Hoechst (**E**,**F**), marked with green arrows.

The treatment of P114 and CMT-U27 mammary cancer cell lines with different concentrations of DOXO for 48 h shows the inhibition of viability in a dose-dependent manner for both canine cell lines. Moreover, the two cell lines have similar values for the half maximal inhibitory concentration with an approximate dose of 13 µM Doxorubicin (Figure 1A,B). Because of the high toxicity of the DOXO, we chose to apply the smallest concentration that shows an inhibitory effect in the cell proliferation in the canine mammary tumor cell lines. Due to the relatively equal concentration of DOXO corresponding to the IC50 of each cell line, we decided to continue the experiments with 2.5 µM of therapeutic agent to compare the response of each cell line under standard conditions.

### 3.2. Doxorubicin Treatment Decreases Cell Viability and Induces Apoptosis in Cell Lines

The triple-fluorescence staining for the cytoskeleton, mitochondria, and nucleus shows damage to the cells through the treatment (Figure 1C,D). The fluorescence microscopy assay revealed that DOXO treatment after 48 h induced in P114 and CMT-U27 cell lines a decreased mitochondrial membrane potential activity and morphological changes, including nuclear DNA condensation, nuclear shrinkage, and fragmentation (marked with green arrows). We can observe a significant reduction in the viable cell’s percentage to 30% (*p*-value = 0.0061) in the P114-treated cells versus untreated cells and 32% for the CMT-U27 cells (*p*-value = 0.0002) (Figure 1E,F).

### 3.3. Reduced Cell Confluency in the Treated versus Untreated Cells

**Figure 2:**
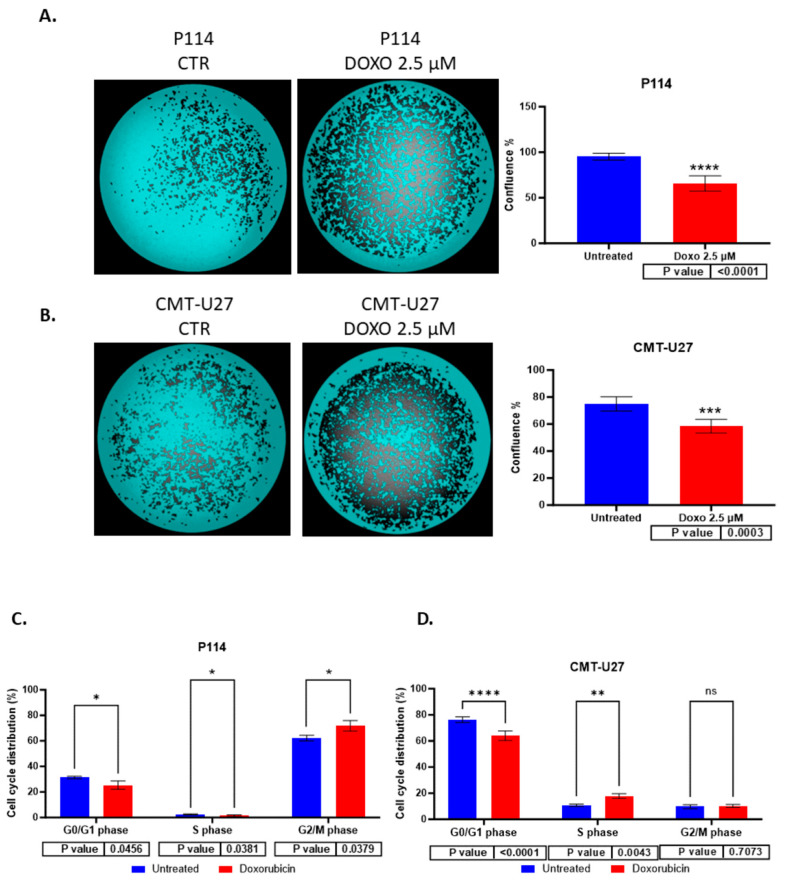

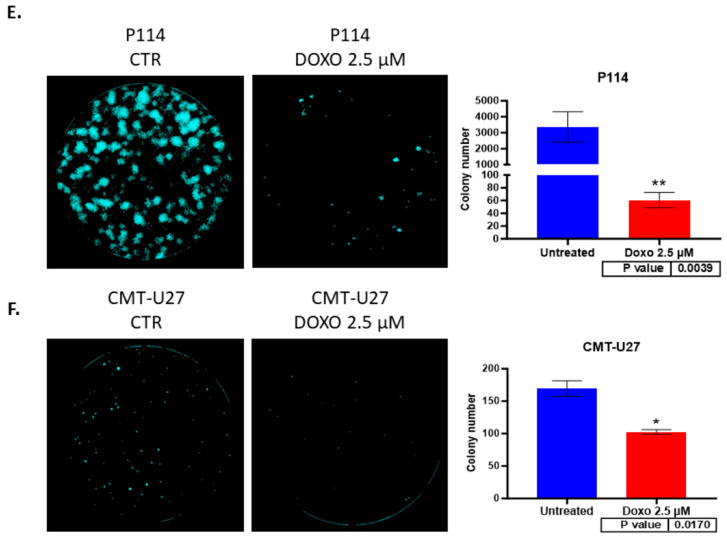
The confluency quantification after 2.5 µM DOXO treatment for 48 h. P114 and CMT-U27 cell lines treated with Doxorubicin show decreased confluency evaluated using Celigo Image Cytometer (**A**,**B**). Cell cycle distribution after 2.5 µM DOXO treatment for 48 h. P114 and CMT-U27 cell lines treated with Doxorubicin show a cell cycle arrest in G0/G1 phase (**C**,**D**). Colony formation after 2.5 µM DOXO treatment for 48 h. P114 and CMT-U27 cell lines treated with Doxorubicin show decreased colony formation capacity. Colony quantification in treated cells versus untreated cells after 7 days using Celigo Image Cytometer (**E**,**F**).

P114 and CMT-U27 treated with 2.5 µM of DOXO for 48 h present a lower confluency rate than untreated cells. We can observe that the treated cells show between a 30 and 40% reduced confluency versus the untreated cells (P114 65.62% and CMT-U27 58.44%) (Figure 2A,B).

### 3.4. Cell Cycle Arrest Induced by Doxorubicin

Doxorubicin induces cell cycle arrest in the G0/G1 phase for the CMT-U27 and in the G2/M for the P114. For the evaluation of cell growth inhibition induced by ABT-199, a cell cycle assay was performed using Celigo imaging flow cytometry. In both cell lines, a decreased percentage of cells was observed in the G0/G1 phase in treated cells versus control cells. In the P114-treated cell line, the S phase distribution was slightly decreased (*p*-value 0.0456), the G2/M phase distribution increased from 62% to 72% (*p*-value 0.0379), and the G0/G1 phase distribution decreased from 31% to 25% (*p*-value 0.0456). CMT-U27-treated cells showed an increased distribution in the S phase from 10% to 17% (*p*-value 0.0043), while the G0/G1 phase distribution decreased from 76% to 64% (*p*-value < 0.0001). In the G2/M phase, no statistically significant changes were observed (Table 1) (Figure 2C,D).

### 3.5. Doxorubicin Reduces Colony Formation in Both Canine Mammary Cancer Cell Lines

Both canine cell lines treated with 2.5 µM of DOXO for 48 h show a significantly reduced colony formation capacity demonstrated through the ability of untreated cells to form more colonies compared to the treated (P114 *p*-value 0.0039; CMT-U27 *p*-value 0.017) ones where we can observe a significantly low number of colonies after 7 days from the treatment (Figure 2E,F).

### 3.6. Doxorubicin Impairs Cell Migration in Both Canine In Vitro Mammary Cancer Models

**Figure 3:**
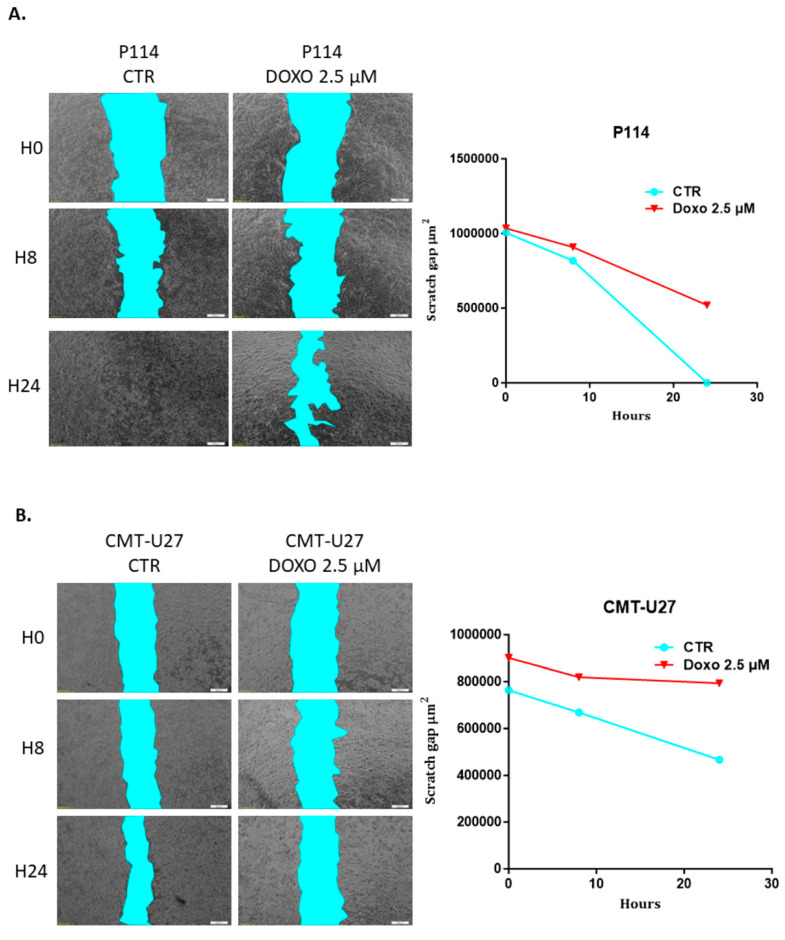

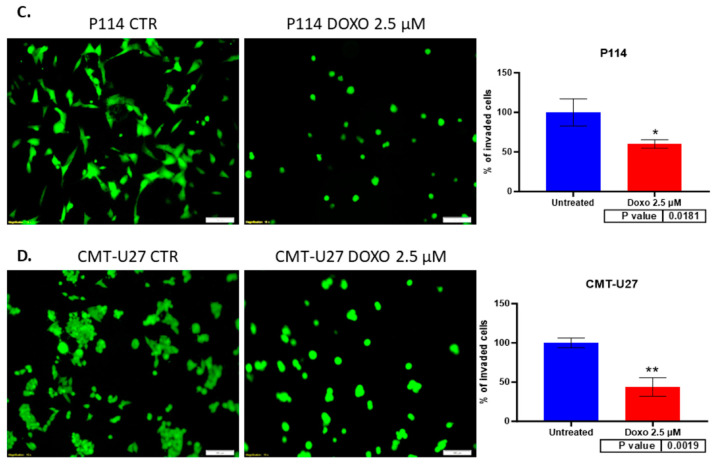
Scratch assay and quantification of cell migration after 2.5 µM DOXO treatment for 48 h. P114 and CMT-U27 cell lines treated with Doxorubicin show decreased migratory capacity after 48 h. Wound area quantification in control cells versus treated ones was evaluated after 0 h, 8 h, and 48 h using an Olympus IX71 microscope, objective 4×, and CellSens Imaging software (**A**,**B**). Invasion capacity evaluation using Matrigel (Invitrogen) and fluorescence microscopy. In both canine cell lines treated with DOXO, we can observe a reduced percentage of invaded cells versus the untreated cells. The fluorescence microscopy evaluation was performed by marking the cells with Calcein-AM and observed using Olympus IX71 inverted fluorescence microscope (image magnification 20×) (**C**,**D**).

CMT-U27 and P114 cell lines treated with 2.5 µM of DOXO for 48 h show a significantly reduced migratory capacity demonstrated through the ability of control cells to close the gap from the scratch assay compared to the treated ones where the gap is still significantly visible after 48 h (Figure 3A,B).

### 3.7. Doxorubicin Treatment Suppresses Cell Invasion

A significantly suppressed invasion ability was observed in both canine mammary cancer cell lines treated with DOXO. Canine cells treated with 2.5 µM DOXO show a reduced percentage of the invaded cells: a 60% *p*-value of 0.018 for P114 and a 43% *p*-value of 0.001 for the CMT-U27 cell line (Figure 3C,D).

### 3.8. Doxorubicin Treatment Induces EMT Gene Expression Level Alteration

**Figure 4:**
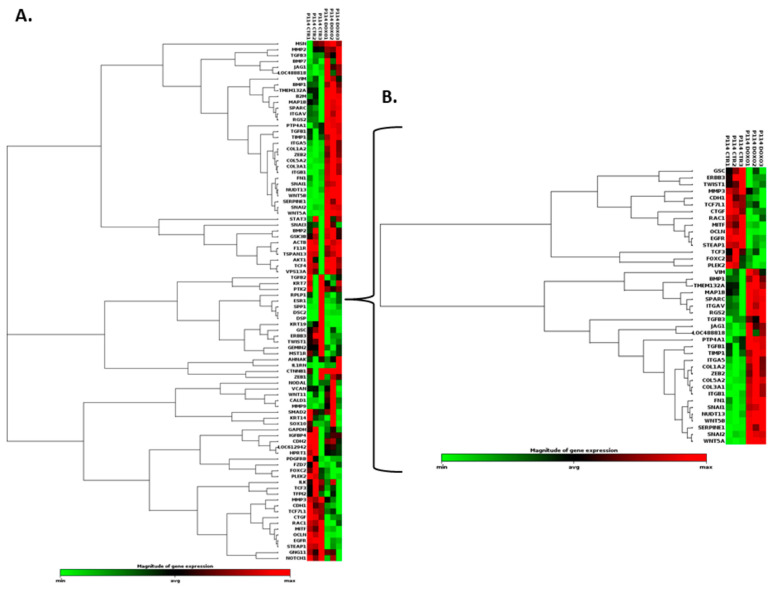

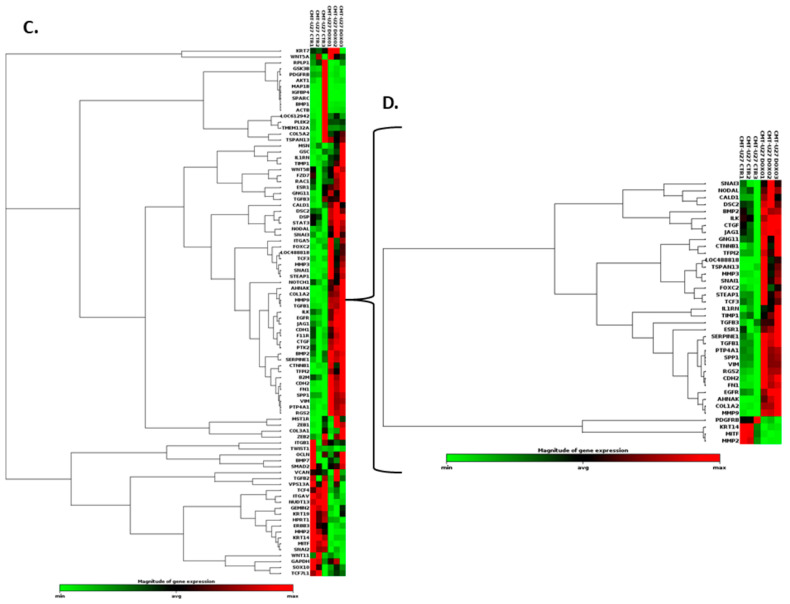

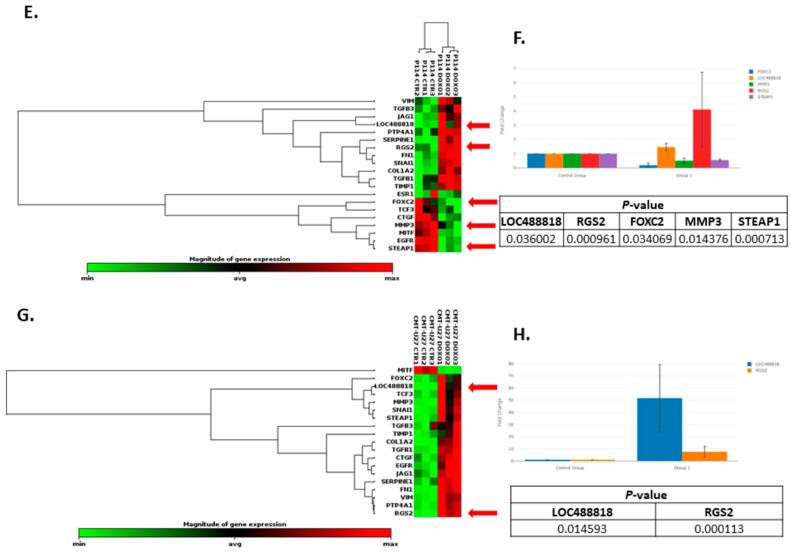
EMT PCR Array gene expression analysis. In both canine cell lines treated with DOXO, we can observe a significantly (*p*-value < 0.05) EMT-related gene expression alteration represented by clustergrams generated using Qiagen analysis software for P114 (**A**,**B**) and CMT-U27 (**C**,**D**). Clustergrams, multigroup plots, and *p*-values for the common altered genes for the treated P114 (**E**,**F**) and CMT-U27 cell lines (**G**,**H**).

**Figure 5:**
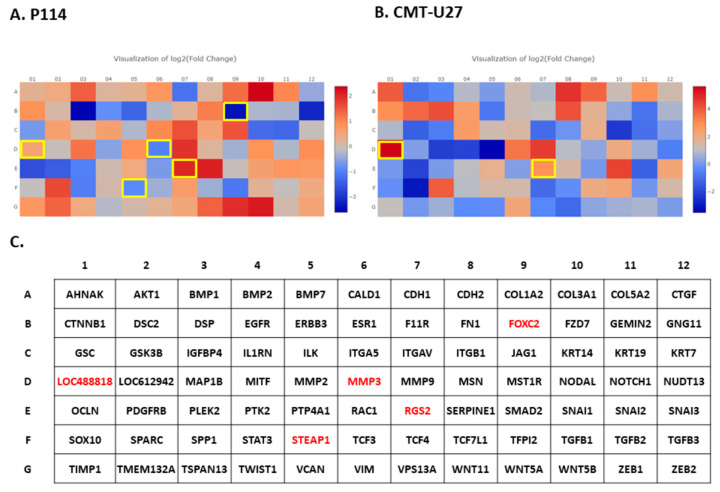
EMT PCR Array gene expression analysis. In both canine cell lines treated with DOXO, we can observe a significant (*p*-value < 0.05) EMT-related gene expression alteration (marked with yellow) represented by heat maps generated using Qiagen analysis software for P114 (**A**) and CMT-U27 (**B**). (**C**) List of the genes from the PCR array and the significantly (*p*-value < 0.05) expressed genes marked with red.

Based on the RT^2^ Profiler PCR Array analysis of Dog Epithelial-to-Mesenchymal-Transition genes, totals of 41 and 38 significantly (*p*-value < 0.05) differentially expressed EMT-related genes for the P114 and CMT-U27 cell lines, respectively, were identified (Figure 4A,B,C,D and Figure 5A,B,C). Table 2 and Table 3 present the fold changes and the list of genes involved in different biological processes including the EMT for both cell lines.

Nineteen genes were identified to be common in the two cell lines treated with DOXO. The P114 cell line treated with DOXO shows an altered expression level for five genes (LOC488818/FGF-BP1, RGS2, FOXC2, STEAP1, and MMP3) and CMT-U27 shows an altered expression level only for two genes (LOC488818 and RGS2) (Figure 4E–H).

## 4. Discussion

The present in vitro study aimed to investigate Doxorubicin’s efficacy in treating CMTs. The obtained results suggest that therapy with Doxorubicin has apoptotic effects on canine in vitro models of mammary cancer and can significantly reduce cell migration, invasion, confluency, and cell colony formation and can induce cell cycle arrest in treated versus untreated cells. Moreover, Doxorubicin treatment can alter EMT gene expression levels. Consistent with the obtained data, DOXO treatment inhibited the cell proliferation at 48 h in two CMT cell lines: one from a primary tumor and one from a lymph node metastasis; furthermore, a substantial effect on the cell cycle was observed, as well as an increase in the gene expression of P-glycoprotein (P-gp) and Breast Cancer Resistance Protein (BCRP) compared to the controls [18].

The epithelial-to-mesenchymal transition (EMT) is a biological process that occurs in various types of tumors, including canine mammary tumors [1]. In this complex cellular phenomenon, epithelial cells are tightly organized and present specific adhesion properties. They suffer molecular and structural characteristics to become more mesenchymal cells, losing their connectivity and acquiring migratory properties [2]. This phenomenon involves cancer progression, invasion, and metastasis [3]. The EMT plays a key role in cancer progression [33,34]. The results suggest that canine mammary tumors can benefit from the activity of Doxorubicin through inhibition of the EMT and different cancer-related biological processes, inducing tumor suppressor mechanisms. Regulator of G protein signaling 2 (RGS2) belongs to a family of proteins that serve as a GTPase-activating protein (GAP) for Gα subunits [35]. It has been described as a potential target in different types of human cancer, including breast cancer [35,36]. Thus, RGS2 was upregulated in MCF7 breast cancer cells, inhibiting cell proliferation [35]. In breast-invasive carcinoma of no special type (BIC-NST), the downregulation of RGS2 was associated with a significantly poorer overall survival rate [35]. LOC488818 is also named FGF-BP1 (Fibroblast Growth Factor Binding Protein 1) and plays an important role in cell proliferation, differentiation, and migration. FGF-BP1 is a protein that acts as a chaperone molecule and is found to be upregulated in breast cancers, squamous cell cancer, and colon cancer [37]. The overexpression of FGF-BP1 was correlated with better prognosis in human breast cancer patients [38]. A transcription factor known as FOXC2, which plays an important role in determining the fate of mesenchymal cells during embryonic development, has also been implicated in cancer cells’ ability to metastasize [39]. FOXC2 is a transcription factor that induces mesenchymal differentiation during the EMT [39,40] and has the potential to be a therapeutic target in the inhibition of metastasis [41]. FOXC2 overexpression was significantly associated with aggressive basal-like human breast cancers [39]. The matrix metalloproteinase (MMP) family of zinc-containing and calcium-dependent proteinases play a crucial role in various biological processes, including tissue remodeling, wound healing, and angiogenesis, and in diseases such as arthritis, cancer, and tissue ulceration. As they hydrolyze extracellular matrix components (ECM), they are also known as matrixins. MMP3 is a zinc-dependent proteolytic enzyme that regulates different biological processes by altering the extracellular matrix [42]. MMP3 over-expression was correlated with tumor growth and metastasis in different types of cancer, including breast cancer [42,43]. Several changes are induced by MMP-3 (increased cell proliferation, apoptosis, angiogenesis, changes in the stroma, etc.), which may result in the development of breast cancer in women. This protein may also affect mammary tumors in canines [44]. STEAP1 is a membrane-bound channel protein found overexpressed in various cancers [45,46,47,48] and was associated with a malignant phenotype and disease prognosis [49]. STEAP1 upregulation significantly inhibited the capacity of migration of the tumor cells and the invasion potential and downregulated the expression of EMT-related genes in human breast cancer [49].

## 5. Conclusions

The treatment and knowledge of canine mammary tumors (CMTs) have made significant progress in veterinary oncology as a spontaneous model for breast cancer research in the last few decades. Using Doxorubicin to target specific biological processes like apoptosis, the cell cycle, migration, invasion, and the EMT may result in better outcomes for patients with this disease. The canine model of breast cancer represents a good opportunity for new therapeutic strategies to be developed for human breast cancer. Doxorubicin is a widely used antineoplastic agent in humans and animals. Multiple mechanisms are involved in DOXO’s action, producing free radicals, intercalation into DNA strands, and inhibiting topoisomerases I and II, causing DNA damage, activating caspases, and ultimately causing apoptosis. The importance of the development of new therapeutic strategies in canine mammary cancer represents a major challenge worldwide. The results of present study provide valuable information for revealing the link between Doxorubicin, phenotypic changes, and proliferation dynamics in malignant cells that may contribute to chemotherapy treatment limitations in the canine model.

## Figures and Tables

**Table 1:** Cell cycle distribution in both canine mammary cancer cell lines.

Cell Line	Mean Number of Cells (%)					
	G0/G1	S	G2/M	G0/G1	S	G2/M
	CTR			DOXO		
P114	31.34	2.47	62.17	25.28	1.37	71.84
CMT-U27	76.35	10.86	9.69	63.95	17.79	10.20

**Table 2:** The list of genes for the P114 cell line treated with Doxorubicin and biological processes.

P114		
Genes	Fold Change (Compared to Control Group)	Biological Processes
BMP1	2.73	EMT, Cell growth and proliferation
CDH1	0.44	EMT
COL1A2	2.43	EMT
COL3A1	5.24	EMT
COL5A2	2.00	EMT
CTGF	0.73	fJAG
EGFR	0.57	Cell growth and proliferation
ERBB3	0.38	Cell growth and proliferation
FN1	2.16	EMT
FOXC2	0.18	EMT, Differentiation and development, Cell growth and proliferation, fJAG, Transcription factors
GSC	0.63	EMT
ITGA5	1.96	EMT
ITGAV	3.04	EMT
ITGB1	1.45	fJAG
JAG1	2.93	Differentiation and development, Cell growth and proliferation, Cell migration and motility
LOC488818	1.46	EMT, Cell growth and proliferation
MAP1B	2.33	Cytoskeleton regulation
MITF	0.68	EMT, Differentiation and development, Transcription factors
MMP3	0.50	EMT, fJAG
NUDT13	1.91	EMT
OCLN	0.32	EMT
PLEK2	0.50	Cytoskeleton regulation
PTP4A1	1.35	Differentiation and development
RAC1	0.64	Cell morphogenesis, Cell migration and motility, Cytoskeleton regulation, fJAG
RGS2	4.12	EMT
SERPINE1	4.26	EMT, fJAG
SNAI1	1.52	EMT, Differentiation and development, Cell morphogenesis
SNAI2	1.75	EMT, Transcription factors
SPARC	3.18	EMT
STEAP1	0.55	EMT
TCF3	0.77	Transcription factors
TCF7L1	0.77	Transcription factors
TGFB1	1.17	Cell morphogenesis, Cell migration and motility, fJAG
TGFB3	1.32	Differentiation and development, Cell growth and proliferation, Cell morphogenesis
TIMP1	1.69	EMT, Cell growth and proliferation, fJAG
TMEM132A	2.66	EMT
TWIST1	0.87	EMT, Differentiation and development, Cell morphogenesis, Transcription factors
VIM	1.18	EMT, Cell migration and motility, Cytoskeleton regulation
WNT5A	3.87	EMT, Differentiation and development, Cell morphogenesis, Signal transduction
WNT5B	4.46	EMT, Differentiation and development, Signal transduction
ZEB2	1.46	Transcription factors

**Table 3:** The list of genes for the CMT-U27 cell line treated with Doxorubicin and biological processes.

CMT-U27		
Genes	Fold Change (Compared to Control Group)	Biological Processes
AHNAK	16.47	EMT
BMP2	1.93	Differentiation and development
CALD1	2.51	EMT, Cell migration and motility
CDH2	28.15	EMT, fJAG
COL1A2	14.38	EMT
CTGF	2.61	fJAG
CTNNB1	7.76	Differentiation and development, Cell morphogenesis, Cell growth and proliferation, fJAG, Transcription factors
DSC2	13.75	fJAG
EGFR	5.92	Cell growth and proliferation
ESR1	2.46	Transcription factors
FN1	18.71	EMT, Cell migration and motility, fJAG
FOXC2	3.41	EMT, Differentiation and development, Cell growth and proliferation, fJAG, Transcription factors
GNG11	1.76	EMT, Signal transduction
IL1RN	6.20	EMT
ILK	2.57	Cell growth and proliferation, fJAG
JAG1	3.76	Differentiation and development, Cell growth and proliferation, Cell migration and motility
KRT14	0.21	Differentiation and development
LOC488818	51.71	EMT, Cell growth and proliferation
MITF	0.25	EMT, Differentiation and development, Transcription Factors
MMP2	0.09	EMT, fJAG
MMP3	11.63	EMT, fJAG
MMP9	24.81	EMT, fJAG
NODAL	4.15	Differentiation and development, Cell growth and proliferation, Cell migration and motility
PDGFRB	0.25	Cell growth and proliferation, Cell migration and motility, Signal transduction
PTP4A1	3.61	Differentiation and development
RGS2	7.56	EMT
SERPINE1	2.90	EMT, fJAG
SNAI1	22.24	EMT, Differentiation and development, Cell morphogenesis
SPP1	16.28	EMT, fJAG
STEAP1	2.83	EMT
TCF3	2.67	Transcription factors
TFPI2	4.10	EMT
TGFB1	4.92	Cell morphogenesis, Cell migration and motility, fJAG
TGFB3	2.79	Differentiation and development, Cell growth and proliferation, Cell morphogenesis
TIMP1	2.23	EMT, Cell growth and proliferation, fJAG
TSPAN13	1.55	EMT
VIM	4.84	EMT, Cell migration and motility, Cytoskeleton regulation

## Data Availability

Data are contained within the article.
